# Quantitative evaluation of pulmonary hypertension using 4D flow MRI: A retrospective study

**DOI:** 10.1016/j.heliyon.2024.e31177

**Published:** 2024-05-17

**Authors:** Hirofumi Koike, Takamasa Nishimura, Minoru Morikawa

**Affiliations:** Department of Radiology, Nagasaki University Graduate School of Biomedical Sciences, Nagasaki University School of Medicine, 1-7-1 Sakamoto, Nagasaki, 852-8501, Japan

**Keywords:** Diagnostic imaging, Magnetic resonance imaging, Pulmonary hypertension, Pulmonary artery

## Abstract

**Background:**

Pulmonary hypertension (PH) is a severe vascular disorder that may affect 50 % of patients with heart failure. Currently, right-sided heart catheterization is required to definitively diagnose PH. However, this method is invasive and thus may not be appropriate for repeated, long-term monitoring of PH patients. This retrospective study's aim was to evaluate whether 4D flow magnetic resonance imaging (MRI) can be used to quantitively measure flow parameters to identify patients with PH.

**Methods:**

The study cohort included 97 patients recruited from a single institution and divided into three groups based on echocardiographic estimate of pulmonary artery systolic pressure (PASP): normal group with PASP<36 mmHg, borderline PH group with PASP of 37–50 mmHg, and PH group with PASP>50 mmHg. 4D flow MRI was used to quantitively assess blood flow and velocity, regurgitation, wall shear stress (WSS) and kinetic energy in the pulmonary artery trunk, right main pulmonary artery, and left pulmonary artery. Two experienced radiologists independently analyzed the MR images, blinded to clinical details.

**Results:**

We found a significant difference in WSS in the pulmonary artery trunk, right main pulmonary artery and left main pulmonary artery among the three patient groups. We also found significant differences in the kinetic energy and average through velocity in the pulmonary artery trunk and right main pulmonary artery, and significant differences in the flow rate in the right main pulmonary artery.

**Conclusion:**

These data suggest that 4D flow MRI can quantitate pulmonary artery flow parameters and distinguish between patients with and without PH.

## 1 Introduction

Pulmonary hypertension (PH) is considered a rare disease, with an estimated prevalence of 15–50 cases/million adults [1,2]. However, PH could affect around 1 % of the global population, up to 10 % of individuals older than 65 years, and at least 50 % of patients with heart failure [3]. PH is defined as a mean pulmonary arterial pressure of 20 mmHg or greater, pulmonary artery wedge pressure of 15 mmHg or less, and pulmonary vascular resistance of 3 Wood units measured by right-sided heart catheterization [4]. However, because this method is invasive, and therefore, difficult to perform repeatedly, noninvasive methods to identify and monitor PH patients are required.

Although echocardiography is a readily available noninvasive tool that is a routine part of PH assessment, it has limitations when compared with right-sided heart catheterization. Echocardiography is limited by poor acoustic windows [5,6], interobserver variability [7], the presence of pulmonary valve stenosis [5,8,9], and the discrepancy between systolic and mean PA pressure [10].

Recently, 3-dimensional (3D) phase-contrast magnetic resonance imaging (MRI) (4D flow MRI) has been developed as a minimally invasive method to quantitatively evaluate blood flow through the heart and large vessels during cardiac cycles [[10], [11], [12], [13], [14]]. Furthermore, the characteristic blood flow in PH can be visualized using 4D flow MRI [15,16]. Therefore, this technique may be a useful noninvasive, nonionizing method to diagnose and monitor PH patients. However, there are limited published data on how 4D flow MRI can quantify blood flow changes in PH patients. Therefore, the goal of this study was to quantify flow parameters using 4D flow MRI, and to make a clear distinction between PH patients and individuals without PH.

## 2 Materials and methods

### 2.1 Patient population

Our institutional review board approved this study and waived the need for written informed consent because the study design was retrospective. From April 2020 to August 2021, consecutive 99 patients underwent echocardiography and cardiac 4D flow MRI in our hospital. The required sample size was calculated to be 66 under the following conditions: effect size (f) = 0.4 (large), α error = 0.05, Power (1 - β) = 0.8, and number of groups = 3. Since the number of patients in the HP group was expected to be small, we collected data 1.5 times the required sample size. Before the 4D flow MRI results were analyzed, patients were divided into three groups (normal group, borderline PH group, and PH group) based on their echo results. Two patients without echo data were excluded from the study cohort. Therefore, this study included 97 consecutive patients in the final cohort.

### 2.2 Echocardiographic estimation of PA pressure

Within 1 week of 4D flow MRI, echocardiographic examination was performed by three experienced cardiologists with more than five years of experience in cardiac echo in our hospital using a Toshiba Artida ultrasound machine (Toshiba Medical Systems Corp., Tochigi, Japan) with 2.5 MHz transducers and results were digitally recorded.

The peak tricuspid regurgitation (TR) gradient is the most commonly used measurement to estimate right ventricular (RV) pressure, which should reflect PA pressure if there is no obstruction to blood flow between the RV and PA. The TR gradient is measured by continuous wave Doppler velocity across the tricuspid valve in line with the regurgitation flow. The modified Bernoulli equation (4 x (velocity of TR)^2^) is used to convert this velocity into a pressure gradient [9]. This gradient represents the difference in pressure between the RV and right atrium and can be used as an estimate of RV systolic pressure [17] when right atrial pressure (normal 5–10 mmHg) is added to the derived gradient. This estimated pulmonary artery systolic pressure (PASP) is used to evaluate the likelihood of a patient having PH. We used PASP to divide our patients into three groups: normal group with PASP<36 mmHg; borderline PH group with PASP of 37–50 mmHg; and PH group with PASP>50 mmHg [18].

### 2.3 MR imaging

4D flow sequence was performed during the usual cardiac MRI sequence, 30 s after gadolinium 0.10 mmol/kg was administrated, immediately before the last late gadolinium enhancement MRI.

Electrocardiographically gated cardiac MR imaging, including phase-contrast (PC) imaging of the pulmonary artery trunk, right main pulmonary artery, and left pulmonary artery, was performed, with the patient in a supine position, using a 3.0-T scanner (MAGNETOM Vida; Siemens, Healthcare, Germany) with a 32-channel cardiac phased-array coil. PC imaging data were acquired in the right ventricular outflow tract orientation while the patient was free breathing. Further protocol parameters were as follows: TR/TE, 43/3.04 ms; flip angle, 15°; velocity encoding (VENC), 1.5–2.0 m/s; spatial resolution, 6 × 1.88 × 1.88 mm^3^; time resolution 19–24 phase/cardiac cycle.

### 2.4 MR imaging analysis

**Fig. 1:**
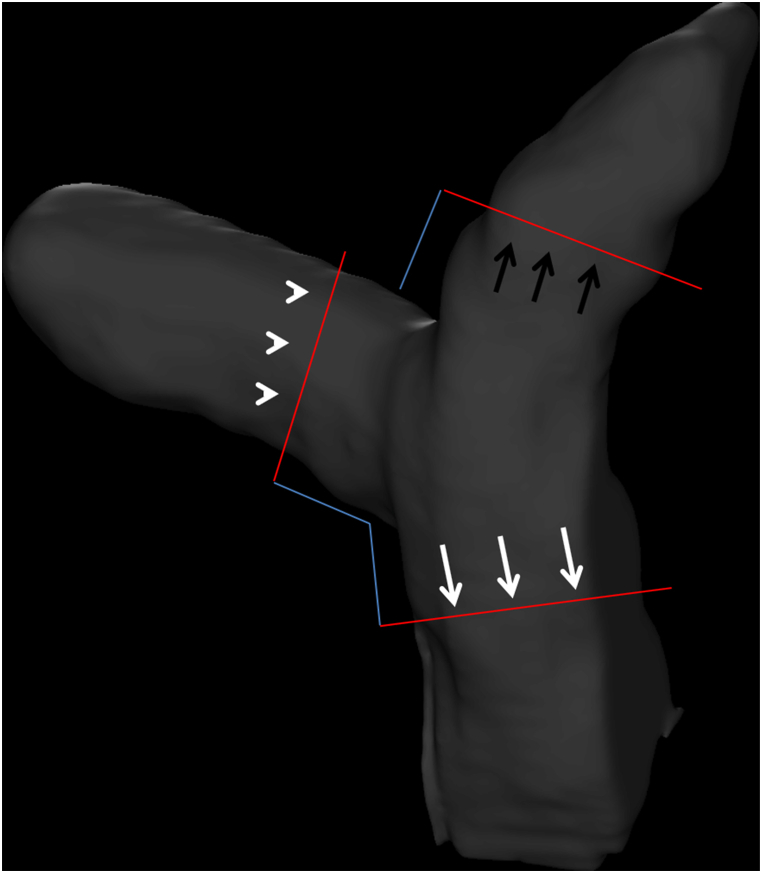
Procession 3D cine image for measuring pulmonary artery flow.

Blood flow volumes were calculated for arbitrary regions within data sets comprising multislice sagittal planes from PC 3 axis cine images, magnitude images, and steady-state free procession cine images obtained from the measurements using iTFlow (Cardio Flow Design Inc., Tokyo, Japan) [19]. Flow volumes were shown with end-diastole as the origin (0 phase). Two experienced radiologists with more than 10 years of experience in cardiac MRI independently analyzed the MR images. The radiologists were blinded to the patients’ clinical conditions and worked independently. Using the procession 3D cine image, a vertical line was drawn on the pulmonary artery trunk 1 cm centrally from the bifurcation of the right pulmonary artery. Also, a vertical line was drawn on the right main pulmonary artery and left main pulmonary artery 1 cm peripherally from the bifurcation of the pulmonary artery trunk (Fig. 1).

A vertical line was drawn on the pulmonary artery trunk 1 cm centrally from the bifurcation of the right pulmonary artery to measure the pulmonary artery trunk (white arrows). A vertical line was drawn on the right main pulmonary artery 1 cm peripherally from the bifurcation of the pulmonary artery trunk to measure the right main pulmonary artery (white arrow heads). A vertical line was drawn on the left main pulmonary artery 1 cm peripherally from the bifurcation of the pulmonary artery trunk to measure the right main pulmonary artery (black arrows).

**Fig. 2:**
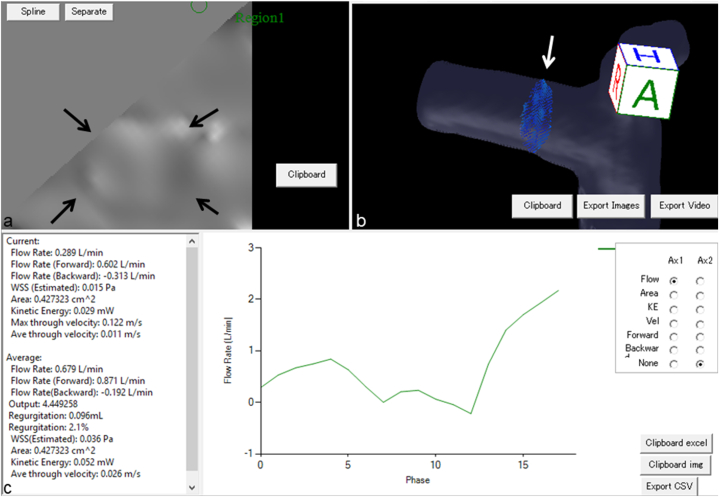
Flow parameters in the right main pulmonary artery in selected cross-sectional area. (a) Axial image shows the right main pulmonary artery in selected cross-sectional area (black arrows). (b) 3D image shows the right main pulmonary artery in selected cross-sectional area (white arrow). (c) Flow parameters were calculated automatically in current and average patterns.

Flow parameters were automatically measured and we investigated average flow parameters in this study (Fig. 2). The mean values measured by two radiologists were used for further analysis.

Flow rate parameters in this study.

Flow rate (ml/min): Flow rate through the cross section.

Forward (L/min): Calculated positive value of the flow rate passing through the cross section.

Backward (L/min): Calculated negative value of the flow rate passing through the cross section.

Output (ml): Total positive flow through the cross section within the imaging time.

Regurgitation (ml): Total negative flow through the cross section within the imaging time.

Regurgitation (%): [Regurgitation (ml)/Output] × 100.

WSS (Pa): Shear force caused by the blood stream acting on a region of the vessel wall. This parameter is becoming increasingly important. It has been shown that regions with a high WSS in the aorta correlate with dysregulation of the extracellular matrix and degeneration of elastic fibers of the ascending aorta [20]. Thus, the WSS can serve not only as a marker for increased stress, and thus a vessel wall location that is particularly at risk, but also as a disease course parameter in known vessel pathology [21].

Area (cm^2^): Cross-sectional area.

Kinetic energy (mW): Energy that must be additionally applied to keep blood flow constant over an area. Increased fluctuation in flow results in a greater pressure loss so that more energy is needed to maintain blood flow. A kinetic energy that is greater than baseline with a similar flow rate means a reduced luminal diameter of the vessel [22]. Kinetic energy quantification is a parameter for determining blood flow efficiency. The higher the kinetic energy, the harder the heart has to work. There is an association between an increased kinetic energy and cardiac disease. Patients with dilated cardiomyopathy have a greater kinetic energy than healthy people [23].

Average through velocity (m/s): Average velocity perpendicular to the surface. It is the simplest parameter, but it is highly significant. Local acceleration of the flow velocity indicates stenosis [24].

### 2.5 Statistical analysis

We used the D'Agostino-Pearson test to assess the normality of the data. We presented non-normally distributed variables as the median range. Quantitative results were expressed as the mean ± the SD or the median range. (The range provided in the tables is the min–max range.)

4D flow parameters were analyzed using the Kruskal-Wallis test and Dunn test with Bonferroni *post hoc* correction. Results were expressed as sensitivity, specificity, and overall accuracy, with 95 % confidence interval (CI) calculated with the normal approximation method [25].

For all tests, a two-sided p-value was used, and differences with a p-value of <0.05 were considered statistically significant. SPSS for Windows, version 24 (SPSS Inc., Chicago, IL, USA) was used for all statistical analyses.

## 3 Results

### 3.1 Patient population in the normal, borderline PH, and PH groups

The patient cohort included 78 normal controls (45 male (57.7 %) and 33 female (42.3 %); mean age (SD): 54.1 years (18.4)), 12 patients with borderline PH (9 male (75 %) and 3 female (25 %); mean age (SD): 59.3 years (14.2)) and 7 patients with PH (7 male (100 %); mean age (SD): 60.3 years (11.8)) (Table 1).

**Table 1:** Patient demographic and clinical characteristics.

Parameter	Normal (n = 78)	Borderline PH (n = 12)	PH (n = 7)
Age, years	54.1 ± 18.4	59.3 ± 14.2	60.3 ± 11.8
Male, n	45	9	7
PASP (mmHg)	24.7 ± 4.8	40.3 ± 2.9	53.9 ± 4.0
History of PH, n	0	0	1
Final diagnosis, n	Unremarkable, 5 DCM, 31 HCM, 8 MI, 10 Myocarditis, 13 Arrhythmia, 6 Cardiac amyloidosis, 2 LVNC, 3	DCM, 5 HCM, 4 Myocarditis, 2 Arrhythmia, 1	DCM, 3 Myocarditis, 1 Cardiac amyloidosis, 2 TOF post operation, 1

Data expressed as n or mean ± standard deviation (range). PH, pulmonary hypertension; PASP, pulmonary artery systolic pressure; DCM, dilated cardiomyopathy; HCM, hypertrophic cardiomyopathy; MI, myocardial infarction; LVNC, left ventricular noncompaction; TOF, Tetralogy of Fallot.

All of these patients were identified in a retrospective review of medical records conducted at a single medical institution.

#### 3.1.1 Pulmonary artery trunk flow

WSS was 0.15 ± 0.08 in the normal group, 0.11 ± 0.05 in the borderline PH group, and 0.10 ± 0.03 in the PH group, showing a significant difference among the three groups (p = 0.035). There was no significant difference between the normal group and the borderline PH group (p = 0.154), the normal group and the PH group (p = 0.174), or the borderline PH and the PH group (p = 1.000).

Kinetic energy was 5.06 ± 3.74 in the normal group, 2.54 ± 1.50 in the borderline PH group, and 1.59 ± 0.88 in the PH group, showing a significant difference among the three groups (p = 0.001). There was a significant difference between the normal group and the borderline PH group (p = 0.047) and between the normal group and the PH group (p = 0.008). In contrast, there was no significant difference between the borderline PH group and the PH group (p = 1.000).

Average through velocity was 0.12 ± 0.06 in the normal group, 0.09 ± 0.03 in the borderline PH group, and 0.09 ± 0.02 in the PH group, showing a significant difference among the three groups (p = 0.039). There was no significant difference between the normal group and the borderline PH group (p = 0.472), the normal group and the PH group (p = 0.073), or the borderline PH and the PH group (p = 1.000, Table 2).

**Table 2:** Flow parameters in the pulmonary artery trunk.

Parameter	Normal (n = 78)	Borderline PH (n = 12)	PH (n = 7)	p-value
Flow rate (ml/min)	3455.8 ± 1284.2	2730.1 ± 727.1	3572.3 ± 866.0	0.134
Forward (L/min)	3.77 ± 1.34	3.04 ± 0.88	3.80 ± 0.90	0.151
Backward (L/min)	−0.31 ± 0.31	−0.31 ± 0.30	−0.20 ± 0.10	0.976
Output (ml)	40.1 ± 19.2	43.9 ± 14.8	39.9 ± 18.0	0.575
Regurgitation (ml)	0.87 ± 1.53	0.69 ± 1.15	0.29 ± 0.70	0.108
Regurgitation (%)	2.27 ± 3.55	1.99 ± 3.96	0.97 ± 2.31	0.121
WSS (Pa)	0.15 ± 0.08	0.11 ± 0.05	0.10 ± 0.03	0.035*
Area (cm^2^)	0.53 ± 0.15	0.59 ± 0.29	0.68 ± 0.16	0.063
Kinetic energy (mW)	5.06 ± 3.74	2.54 ± 1.50	1.59 ± 0.88	0.001*
Ave through velocity (m/s)	0.12 ± 0.06	0.09 ± 0.03	0.09 ± 0.02	0.039*

Data expressed as n or mean ± standard deviation (range). PH, pulmonary hypertension; WSS, wall shear stress; Ave, average.

### 3.2 Right main pulmonary artery flow

Flow rate was 1542.5 ± 654.5 in the normal group, 1278.0 ± 329.7 in the borderline PH group, and 948.9 ± 480.1 in the PH group, showing a significant difference among the three groups (p = 0.019). There was a significant difference between the normal group and the PH group (p = 0.025). In contrast, there was no significant difference between the normal group and the borderline PH group (p = 0.638) or between the borderline PH group and the PH group (p = 0.505).

WSS was 0.19 ± 0.15 in the normal group, 0.13 ± 0.06 in the borderline PH group, and 0.06 ± 0.03 in the PH group, showing a significant difference among the three groups (p = 0.002). There was also a significant difference between the normal group and the PH group (p = 0.002). In contrast, there was no significant difference between the normal group and the borderline PH group (p = 0.672) or between the borderline PH group and the PH group (p = 0.130).

Kinetic energy was 2.33 ± 2.64 in the normal group, 0.94 ± 0.72 in the borderline PH group, and 0.39 ± 0.26 in the PH group, showing a significant difference among the three groups (p < 0.001). There was a significant difference between the normal group and the borderline PH group (p = 0.043) and between the normal group and the PH group (p = 0.002). In contrast, there was no significant difference between the borderline PH and the PH group (p = 0.635).

Average through velocity was 0.10 ± 0.06 in the normal group, 0.07 ± 0.03 in the borderline PH group, and 0.07 ± 0.07 in the PH group, showing a significant difference among the three groups (p = 0.002). There was a significant difference between the normal group and the PH group (p = 0.002). In contrast, there was no significant difference between the normal group and the borderline PH group (p = 0.485) or between the borderline PH group and the PH group (p = 0.182, Table 3).

**Table 3:** Flow parameters in the right main pulmonary artery.

Parameter	Normal (n = 78)	Borderline PH (n = 12)	PH (n = 7)	p-value
Flow rate (ml/min)	1542.5 ± 654.5	1278.0 ± 329.7	948.9 ± 480.1	0.019*
Forward (L/min)	1.76 ± 0.67	1.46 ± 0.33	1.29 ± 0.44	0.049*
Backward (L/min)	−0.23 ± 0.17	−0.18 ± 0.13	−0.34 ± 0.21	0.140
Output (ml)	20.6 ± 9.7	20.9 ± 7.5	9.1 ± 2.7	0.001*
Regurgitation (ml)	0.77 ± 0.90	0.79 ± 0.67	2.05 ± 2.61	0.602
Regurgitation (%)	4.85 ± 8.66	3.59 ± 2.37	14.7 ± 16.1	0.212
WSS (Pa)	0.19 ± 0.15	0.13 ± 0.06	0.06 ± 0.03	0.002*
Area (cm^2^)	0.29 ± 0.11	0.33 ± 0.13	0.40 ± 0.09	0.046*
Kinetic energy (mW)	2.33 ± 2.64	0.94 ± 0.72	0.39 ± 0.26	<0.001*
Ave through velocity (m/s)	0.10 ± 0.06	0.07 ± 0.03	0.07 ± 0.07	0.002*

Data expressed as n or mean ± standard deviation (range). PH, pulmonary hypertension; WSS, wall shear stress; Ave, average.

### 3.3 Left main pulmonary artery flow

Backward was −0.11 ± 0.12 in the normal group, −0.10 ± 0.15 in the borderline PH group, and −0.03 ± 0.02 in the PH group, showing a significant difference among the three groups (p = 0.019). There was a significant difference between the normal group and the PH group (p = 0.018). In contrast, there was no significant difference between the normal group and the borderline PH group (p = 1.000) or between the borderline PH group and the PH group (p = 0.253).

WSS was 0.20 ± 0.10 in the normal group, 0.15 ± 0.06 in the borderline PH group, and 0.13 ± 0.04 in the PH group, showing a significant difference among the three groups (p = 0.015). There was no significant difference between the normal group and the borderline PH group (p = 0.143), the normal group and the PH group (p = 0.061), or the borderline PH and the PH group (p = 1.000, Table 4).

**Table 4:** Flow parameters in the left main pulmonary artery.

Parameter	Normal (n = 78)	Borderline PH (n = 12)	PH (n = 7)	p-value
Flow rate (ml/min)	1608.6 ± 573.0	1444.6 ± 535.2	1875.5 ± 778.0	0.556
Forward (L/min)	1.72 ± 0.58	1.55 ± 0.52	1.94 ± 0.77	0.605
Backward (L/min)	−0.11 ± 0.12	−0.10 ± 0.15	−0.03 ± 0.02	0.019*
Output (ml)	21.9 ± 9.2	23.5 ± 9.2	18.0 ± 4.7	0.366
Regurgitation (ml)	0.18 ± 0.35	0.18 ± 0.37	0.14 ± 0.26	0.899
Regurgitation (%)	1.13 ± 2.94	2.57 ± 7.34	0.62 ± 1.08	0.886
WSS (Pa)	0.20 ± 0.10	0.15 ± 0.06	0.13 ± 0.04	0.015*
Area (cm^2^)	0.26 ± 0.07	0.29 ± 0.10	0.36 ± 0.08	0.012*
Kinetic energy (mW)	1.52 ± 1.56	0.82 ± 0.85	0.76 ± 0.59	0.088
Ave through velocity (m/s)	0.11 ± 0.04	0.08 ± 0.03	0.08 ± 0.02	0.074

Data expressed as n or mean ± standard deviation (range). PH, pulmonary hypertension; WSS, wall shear stress; Ave, average.

### 3.4 Interobserver agreement in flow parameters

Interobserver agreement regarding Flow, Forward, Backward, Output, Regurgitation (ml), Regurgitation (%), WSS, Area, Kinetic energy and Average through velocity on the pulmonary artery trunk, right main pulmonary artery and left main pulmonary artery was excellent (Table 5).

**Table 5:** Interobserver agreement in flow parameters.

Parameter	ICC values in pulmonary artery trunk	ICC values in right main pulmonary artery	ICC values in left main pulmonary artery
Flow rate (ml/min)	0.968 [95 % CI, 0.948–0.980]	0.950 [95 % CI, 0.927–0.966]	0.943 [95 % CI, 0.916–0.962]
Forward (L/min)	0.978 [95 % CI, 0.959–0.987]	0.946 [95 % CI, 0.920–0.963]	0.945 [95 % CI, 0.919–0.963]
Backward (L/min)	0.921 [95 % CI, 0.884–0.946]	0.934 [95 % CI, 0.900–0.956]	0.808 [95 % CI, 0.726–0.867]
Output (ml)	0.962 [95 % CI, 0.940–0.975]	0.957 [95 % CI, 0.937–0.971]	0.949 [95 % CI, 0.925–0.965]
Regurgitation (ml)	0.874 [95 % CI, 0.818–0.914]	0.876 [95 % CI, 0.821–0.915]	0.849 [95 % CI, 0.782–0.896]
Regurgitation (%)	0.838 [95 % CI, 0.767–0.888]	0.864 [95 % CI, 0.803–0.907]	0.833 [95 % CI, 0.760–0.885]
WSS (Pa)	0.959 [95 % CI, 0.936–0.973]	0.977 [95 % CI, 0.966–0.985]	0.899 [95 % CI, 0.851–0.931]
Area (cm^2)^	0.949 [95 % CI, 0.886–0.973]	0.947 [95 % CI, 0.914–0.966]	0.861 [95 % CI, 0.787–0.909]
Kinetic energy (mW)	0.958 [95 % CI, 0.939–0.972]	0.983 [95 % CI, 0.975–0.989]	0.964 [95 % CI, 0.947–0.976]
Ave through velocity (m/s)	0.963 [95 % CI, 0.945–0.975])	0.978 [95 % CI, 0.968–0.985]	0.927 [95 % CI, 0.891–0.951]

ICC, intraclass correlation coefficient; CI, confidence interval; WSS, wall shear stress; Ave, average.

**Fig. 3:**
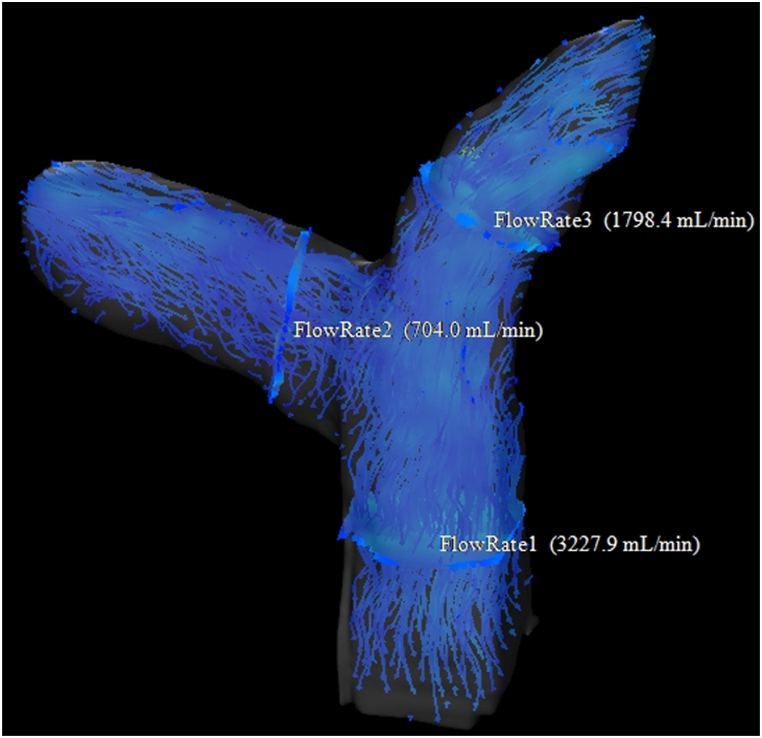
Image for a 52-year-old man with pulmonary hypertension (PH). PASP was 56 mmHg. This 4D flow map shows decreased blood flow in the pulmonary artery trunk, right main pulmonary artery, and left main pulmonary artery.

**Fig. 4:**
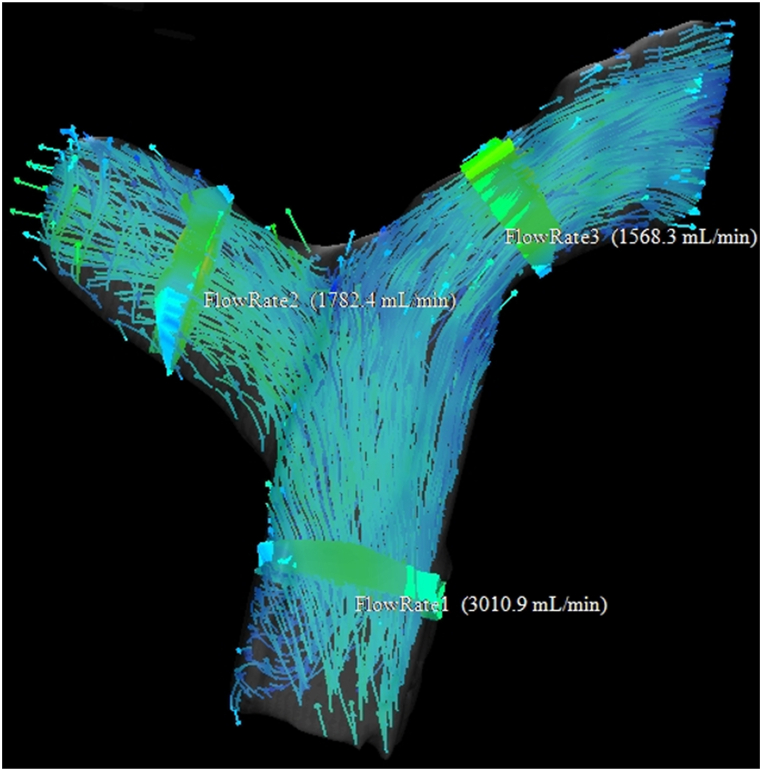
Image for a 59-year-old man without PH. PASP was 27 mmHg. This 4D flow map shows blood flow is maintained in the pulmonary artery trunk, right main pulmonary artery, and left main pulmonary artery.

Fig. 3 shows 4D flow map imaging in a patient with PH. This shows decreased blood flow in the pulmonary artery trunk, right main pulmonary artery, and left main pulmonary artery. In comparison, Fig. 4 shows 4D flow map imaging in a patient without PH. This shows that blood flow is maintained in the pulmonary artery trunk, right main pulmonary artery, and left main pulmonary artery.

## 4 Discussion

In this study, we used 4D flow MRI to identify a significant difference in WSS in the pulmonary artery trunk, right main pulmonary artery, and left main pulmonary artery between the patients in the normal group, borderline PH group, and PH group. We also found a significant difference in the kinetic energy and average through velocity in the pulmonary artery trunk and right main pulmonary artery, and significant differences in the flow rate, Forward, and Output in the right main pulmonary artery among the three groups. Since PH causes alterations in pulmonary artery blood flow [[26], [27], [28], [29], [30], [31]], we speculate that these flow parameters may decrease with PH.

The right main pulmonary artery showed significant differences in more flow parameters than the other anatomical locations, and thus appears to be strongly influenced by PH. The pulmonary artery trunk arises from the right ventricular outflow tract and courses posteriorly and superiorly to the left of and posterior to the aorta [32]. The right main pulmonary artery is within the pericardium for more than three-quarters of its length and runs horizontally to the right behind the ascending aorta and superior vena cava. In contrast, the main left pulmonary artery passes inferiorly and posteriorly and exits the pericardium below the aortic arch at the ligamentum arteriosum. It arches over and behind the left mainstem bronchus and curves around three-quarters of the circumference of the left upper lobe bronchus [33]. Since the right main pulmonary artery runs horizontally and is located perpendicular to the right lung, it may be influenced by increased peripheral pulmonary vascular resistance in PH. Moreover, the right main pulmonary artery is located perpendicular to the pulmonary artery trunk, so its blood flow may be smooth and be affected by only PH. In contrast, the trajectory of the left main pulmonary artery is curved, so this may be less affected by PH than the right main pulmonary artery. There was a significant difference in Backward in the left pulmonary artery among the three groups. This may be because of the pulmonary arteriosclerosis that occurs in PH, which reduces Backward blood flow of the left pulmonary artery during cardiac contraction.

4D flow MRI is less invasive than right sided heart catheterization. With 4D flow MRI, we can directly and quantitatively evaluate pulmonary artery blood flow.

The results of this study should be clinically useful because with noninvasive quantitative evaluation of blood flow parameters, we may be able to diagnose patients with PH, and monitor response to treatment. However, it seems necessary to consider how to use echocardiography and 4D flow MRI for different patients. For example, it may be useful to perform 4D flow MRI in patients who are difficult to evaluate using echocardiography, especially those who are obese, or in patients who need to evaluate the left and right pulmonary arteries separately, such as during pulmonary angioplasty or pulmonary artery thrombectomy.

This study had several limitations. First, this study included a small number of PH patients from a single institution, and they were included in the study based on echocardiography results. Therefore, the study lacked an external validation cohort. In addition, differences may occur when data from this study are analyzed in other applications. Second, all patients in this study underwent cardiac MRI with 4D flow sequence because of suspected heart disease. Therefore, the results generated in this population subset cannot be generalized to the entire population. Thirdly, 4D flow data analysis was done manually and only one sectional measurement was performed for each of the pulmonary artery trunk, right main pulmonary artery, and left main pulmonary artery. Finally, the gold standard for evaluating PH is cardiac catheterization, and previous reports have compared cardiac catheterization and 4D flow MRI for evaluating PH. The comparison between echocardiography and 4D flow MRI has not been clear, and further investigation is needed to detect specific reference values for sensitivity and specificity between echocardiography and 4D flow MRI.

## 5 Conclusions

4D flow MRI identified significant differences in flow parameters, particularly in the right pulmonary artery, between the normal, borderline PH, and PH groups. Therefore, 4D flow MRI may be a new, noninvasive method to evaluate PH patients.

## Funding

There are no funding resources.

## Disclosure of interest

The authors report no relationships that could be construed as a conflict of interest.

## Ethics and consent

The study was approved by the Institutional Review Board of the Nagasaki University Hospital (The date of approval (16/9/2020) and the project identification code (20091416)).

## CRediT authorship contribution statement

**Hirofumi Koike:** Writing – review & editing, Writing – original draft, Resources, Methodology, Investigation, Formal analysis, Data curation, Conceptualization. **Takamasa Nishimura:** Validation, Resources, Formal analysis. **Minoru Morikawa:** Writing – review & editing, Software, Project administration, Funding acquisition.

## Declaration of competing interest

The authors declare the following financial interests/personal relationships which may be considered as potential competing interests:There are no additional relationships or activities to declare. If there are other authors, they declare that they have no known competing financial interests or personal relationships that could have appeared to influence the work reported in this paper.
